# Study on the influencing factors of ecological spatial grouping optimization in the huangshan region based on entropy weight topsis

**DOI:** 10.1371/journal.pone.0329956

**Published:** 2025-08-26

**Authors:** Le Zhang, Qiqi Rao, Mingxia Yuan, Yanlong Guo

**Affiliations:** 1 Anhui Cultural Tourism Innovative Development Research Institute, Anhui Jianzhu University, Hefei, China; 2 Anhui Institute of Contemporary Studies, Anhui Academy of Social Sciences, Hefei, China; Gonbad Kavous University, IRAN, ISLAMIC REPUBLIC OF

## Abstract

This study evaluates and analyzes the influencing factors of ecological spatial configuration optimization in the Greater Huangshan Region. By identifying and assessing these factors, it aims to reveal the mechanisms underlying ecological space optimization in the area. The findings provide a theoretical foundation for promoting high-quality development and optimizing ecological spatial governance policies in the Greater Huangshan Region.An index system for ecological space configuration optimization in the Greater Huangshan Region was constructed based on the DPSIR model, and the Entropy Weight-TOPSIS method was applied to evaluate the ecological space from 2018 to 2023. From a temporal dimension, the configuration optimization of ecological space in the Greater Huangshan Region showed an upward trend from 2018 to 2023, with a significant improvement in ES levels. From a spatial dimension, there are certain differences in the group optimization level of ecological space among cities in the region, with the spatial distribution generally showing the characteristics of Anqing > Huangshan > Xuancheng > Chizhou. Although the group optimization of ES in the Greater Huangshan Region has significantly strengthened from 2018 to 2023, the ecological situation remains severe. It is necessary to accelerate socioeconomic development while actively implementing various ecological protection policies, comprehensively improving the group optimization level of ecological space in each city, and thus promoting high-quality development in the Greater Huangshan Region.

## 1. Introduction

Under the backdrop of rapid globalization, humanity's demand for resources continues to grow exponentially, with human activities progressively extending into natural ecological areas [1]. Accelerated urbanization poses severe threats to natural environments, leading to air pollution, biodiversity decline, and reduction of natural habitats [2]. Concurrently, large-scale production activities have weakened the structure and functions of urban ecosystems, intensifying conflicts between urban ecology, agriculture, and construction spaces [3–5].For developing countries like China, this situation is particularly acute [6]. Despite sustained rapid economic growth, ecological conditions remain concerning—approximately 20% of soil suffers erosion, while about 1 billion mu (666,667 km²) of grassland has degraded [7]. With growing public awareness of ecological space safety and quality, balancing urbanization with ecological conservation has become an urgent challenge in urban planning [8,9]. The Chinese government proposed in 2019 the need to comprehensively consider population, economic structure, land use, and ecological factors to scientifically arrange “production-living-ecological” (ES) spatial layouts [10].

Ecological space carries dual connotations: In its narrow sense, it refers to the capacity of natural and semi-natural ecosystems to maintain healthy and stable states, emphasizing self-regulation and sustainable outputs (such as forest carbon sequestration and wetland purification). Broadly defined, it focuses on human adaptation to dynamic ecosystem services, highlighting coordinated development between social systems and ecosystems (e.g., mitigating urban heat islands through ecological corridor planning).

Early research defined urban ES mainly as green spaces centered around public parks [11], later expanding to include forests, grasslands, farmlands, and incorporating concepts like “blue-green spaces” and green infrastructure [12–15]. As critical green infrastructure, ES plays vital roles in climate regulation (alleviating heat island effects), flood prevention, air/water purification, and biodiversity conservation [16], serving as fundamental elements for urban ecological security and sustainable development [17]. Currently, ES-related research has become a key academic focus area [18].

Current research focuses on two main aspects: First, land carrying capacity within ecological space serves as the foundation for configuration optimization [19,20]. This involves evaluating resource supply, environmental benefits (ecological capacity), economic development (production capacity), and social development (living capacity) potentials to guide land use planning [21,22]. The essence of Configuration Optimizationlies in establishing quantitative indicator systems to scientifically assess regional ecological baselines and environmental conditions, then formulating adaptive optimization strategies through spatial configuration adjustments aligned with development needs.

For instance, scholars conducted case studies in China's Three Gorges Reservoir area to examine the relationship between construction land expansion and ecological environment evolution, generating future projections based on current research trends [23]. Second, researchers emphasize suitability assessments for spatial functions within specific regions [24,25]. Cui et al. [26] established ecological buffer zones in resource-deficient areas to expand ES scale while providing ecological services for humans and organisms. Liao et al. [27] developed a novel ES optimization method integrating ESV, BP-ANN, and CLUE-S models, effectively controlling agricultural productivity decline while enhancing ecological functions like soil/water conservation and water purification. Huang et al. [28] proposed expanding existing ES areas while improving ecological quality through coordinated economic-ecological development. However, most current ES-GO studies concentrate on national/regional scales, limiting their applicability in guiding urban cluster-level ecological protection, restoration, and planning.Methodologically, ES research predominantly employs “Pressure-State-Response (PSR) models” or “ES environmental indicators” to establish multidimensional quantitative evaluation systems for qualitative analysis.

The classic PSR framework comprises three components: Pressure, State, and Response. The DPSIR model, developed by the European Environment Agency (EEA), integrates advantages from both PSR and “Driving Force-State-Response" (DSR) models [29,30]. Some scholars have enhanced PSR models by incorporating ecosystem dynamics and extracting high-quality driving factors from 2000–2017 multi-source remote sensing data, achieving high-precision spatiotemporal monitoring [31]. ES environmental index assessments typically involve three steps: ecological indicator selection, weight calculation, and composite index construction [32]. For example, Zhang et al. used vegetation/landscape indices as predictors and surface temperature as response variable, demonstrating gray-green spaces’ critical role in mitigating heat islands and improving thermal comfort, thereby informing urban planning strategies [33]. However, current evaluation systems require subjective weight determination for models and ecological indicators, hindering objective environmental quality assessment.

Existing research shows two main limitations: First, the lack of urban cluster-level studies reduces practical guidance value for ecological protection and planning. Second, subjective weight assignments in evaluation systems challenge comprehensive and objective environmental change assessments.

To address these gaps, this study selects the Greater Huangshan Region (DHR) as the research area, with two primary objectives: (1) Develop a comprehensive index system based on the Driving Force-Pressure-State-Impact-Response (DPSIR) model to evaluate ES-GO influencing factors through analysis of DHR's ES development trends and practical issues. (2) Apply entropy weight-TOPSIS method for objective weighting to minimize subjective bias, ensuring more reliable and practical evaluation outcomes.

## 2. Study area, data sources, and methodology

### 2.1. Study area

**Fig 1:**
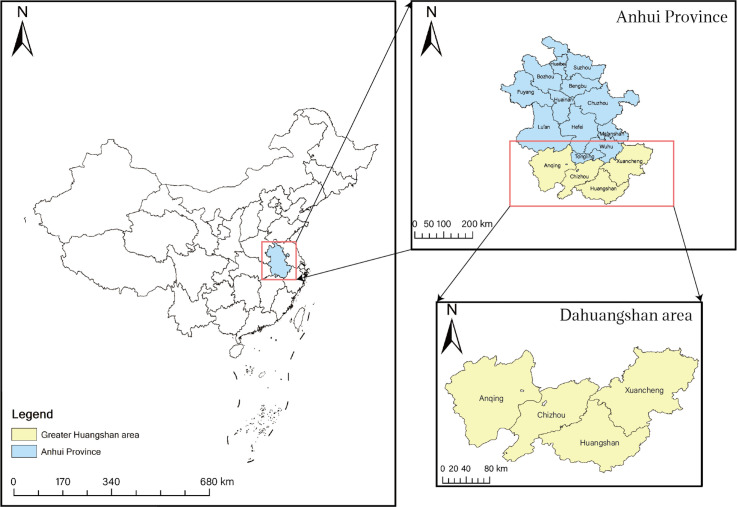
Schematic map of the study area.

The “Greater Huangshan Region" (GHR) represents a strategic cultural-tourism initiative in Anhui Province and the Yangtze River Delta. Covering 44,000 km² across 18 counties in Huangshan, Chizhou, Anqing, and Xuancheng cities, this area hosts 9.3 million residents and concentrates 227 A-rated scenic spots (one of China's eight major tourist clusters), including iconic mountains like Huangshan, Qiyunshan, Jiuhuashan, and Tianzhushan, as shown in Fig 1. It preserves five national historical cities and over 9,000 ancient villages.Following provincial government directives in 2024 to prioritize Greater Huangshan development for tourism enhancement, the region faces dual pressures: intensive tourism development straining water/vegetation resources, and escalating waste/water pollution from mass tourism. These conditions create a representative case study for balancing tourism growth with ecological space protection. As a model zone for regional coordination and cultural-tourism integration, this area offers both unique characteristics and universal research value for developing replicable ES configuration optimization frameworks.

### 2.2. Data sources

All original data involved in this study are derived from official statistical sources, including 31 indicators in aspects such as population, economy, society, resources, and environment. These primarily come from the Anhui Statistical Yearbook, Huangshan Statistical Yearbook, Anqing Statistical Yearbook, Chizhou Statistical Yearbook, and Xuancheng Statistical Yearbook from 2018 to 2023. For missing indicators in individual years, the linear interpolation method was used to supplement and complete the data.

### 2.3. Methodology

#### 2.3.1. DPSIR model.

**Fig 2:**
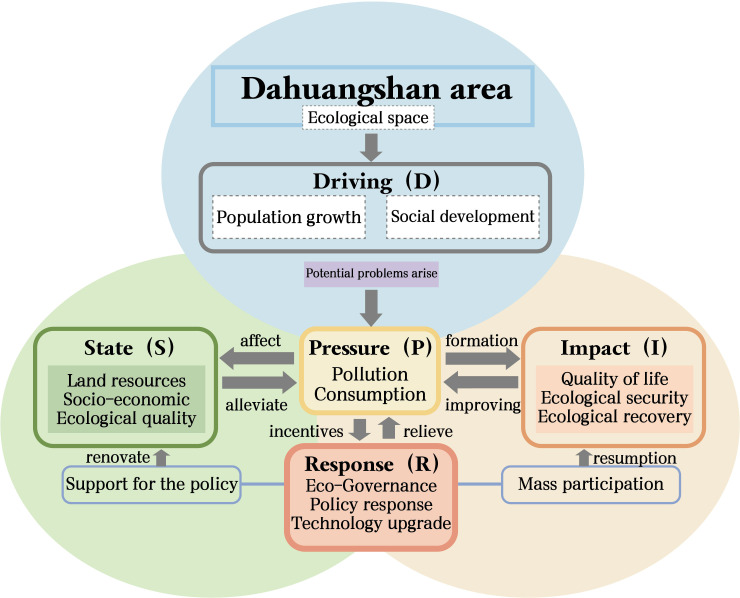
DPSIR model framework.

This framework comprises five subsystems: Driving Force (D), Pressure (P), State (S), Impact (I), and Response (R), with interrelationships shown in Fig 2.

Specifically, the Driving Force (D) primarily stems from socioeconomic development. Pressure (P) identifies the environmental, social, and economic consequences of human activities such as resource extraction/utilization, climate change, and land use patterns. State (S) reflects the current conditions and evolving trends of environmental subsystems (e.g., water, air, and soil). Impact (I) describes how these system states affect both natural and social environments. Response (R) represents policy actions implemented to mitigate pressures and improve environmental quality [35].Compared to PSR and DSR frameworks, the DPSIR model offers enhanced capabilities for systematically analyzing causal relationships between human activities and ecological space environments [36]. This approach enables more scientifically grounded indicator selection and delivers comprehensive evaluation outcomes [37]. Currently, DPSIR has become a widely adopted methodology for investigating urban ecological security [38], spatiotemporal patterns of land expansion [39], marine ecosystem assessments [40], environmental studies [41], and land use planning [42].

#### 2.3.2. Indicator system development.

Building upon domestic and international evaluation studies [42–46], this research first analyzes the actual conditions of the Greater Huangshan Region. Subsequently, considering regional ecological space development trends and adhering to principles of non-overlapping, non-redundant, and measurable indicators, we established a three-tier indicator system integrated with the DPSIR model.Indicators should cover the “nature-economy-society” composite system to avoid single-dimensional bias. Meanwhile, when selecting indicators, those with long-term continuous monitoring data should be prioritized.First Tier: Objective Tier.Measures the configuration optimization influencing factors of ecological space in the evaluated urban cluster.Second Tier: Criterion Tier.Incorporates five subsystems: Driving Force, Pressure, State, Impact, and Response.Third Tier: Indicator Tier.Comprises 31 specific foundational metrics (Table 1).

**Table 1:** DPSIR-based indicator system for ES-GO influencing factors in the Greater Huangshan Region.

Objective Layer	Criterion Layer	Indicator Layer	References	Unit	Weight	Indicator Nature
Study on Factors Influencing Ecological Space Configuration Optimization in the Greater Huangshan Region	Driving Force (D)	D1 Population Growth Rate	[34] Nathwani J,Lu X,Wu C, et al.	‰	0.0350	+
		D2 Urbanization Rate		%	0.0237	+
		D3 Regional Gross Domestic Product (GDP)		100 million yuan	0.0247	+
		D4 Urban Per Capita Disposable Income		yuan	0.0170	+
		D5 General Public Budget Revenue		100 million yuan	0.0303	+
		D6 Urban Construction Land Area		square kilometers	0.0164	+
	Pressure (P)	P1 Electricity Consumption Across Society	[35] Wei Y,Zhu X,Li Y, et al	100 million kWh	0.0270	–
		P2 Private Vehicle Ownership		vehicles	0.0295	–
		P3 Annual Number of Domestic and International Tourists Received		10,000 persons	0.0267	–
		P4 Number of Geological Disasters Occurred		times	0.0565	–
		P5 Annual Total Water Supply		10,000 tons	0.0325	–
		P6 Annual Total Natural Gas Consumption		10,000 tons	0.0325	–
	State (S)	S1 Annual Precipitation	[36] Ruidong Z,Chuanglin F,Haimeng L, et al.	square meters	0.0455	+
		S2 Annual Average Temperature		millimeters	0.0449	+
		S3 Sown Area of Food Crops		degrees Celsius	0.0228	–
		S4 Per Capita Urban Road Area		thousand hectares	0.0302	+
		S5 Total Water Resources		100 million cubic meters	0.0411	+
		S6 Total Afforestation Area		hectares	0.0243	+
	Impact (I)	I1 Urban Air Quality Compliance Rate	[37] Xinguang L,Jun Z,Tong L, et al.	%	0.0334	+
		I2 Annual Average Concentration of Fine Particulate Matter (PM2.5)		micrograms	0.0469	–
		I3 Average Urban Road Traffic Noise Level		decibels	0.0272	–
		I4 Number of Parks in Each City		units	0.0332	+
		I5 Forest Coverage Rate		%	0.0262	+
		I6 Green Coverage Area		hectares	0.0213	+
		I7 Public Utility Land		square kilometers	0.1028	+
	Response (R)	R1 Added Value of Tertiary Industry	[38] Guangming Y,Qingqing G,Junyue L, et al.	100 million yuan	0.0244	+
		R2 Urban Sewage Treatment Rate		%	0.0322	+
		R3 Number of Students Enrolled in Regular Higher Education Institutions		persons	0.0258	+
		R4 Nature Reserves		units	0.0185	+
		R5 Amount of Domestic Waste Harmlessly Treated		10,000 tons	0.0212	+
		R6 Per Capita Urban Park Green Space Area		square meters	0.0267	+

#### 2.3.3. Entropy weight TOPSIS metho.

TOPSIS is a common multi-criteria evaluation method first proposed by Yoon & Hwang. The model aims to compare multi-dimensional and multi-index indicators, ranking evaluation objects based on their distances to the ideal solution and negative ideal solution [47]. However, the traditional TOPSIS method mainly relies on subjective weight assignment by experts, which may cause evaluation results to deviate from reality. The entropy weight method, by contrast, is an objective weight assignment method that determines weights based on the inherent data information characteristics of samples. In view of this, this study combines the advantages of the entropy weight method and TOPSIS [48]. First, indicator weights are determined using the entropy weight method, and then TOPSIS is used to calculate the influence factor index for ecological space configuration optimization in the Greater Huangshan region [49]. The specific steps are as follows.

(1) Data Normalization Processing:

The extremum method is used to standardize the data of each evaluation indicator, eliminating the influence of dimensionality and order of magnitude among the indicators in the evaluation index system for factors influencing ecological space configuration optimization in the Greater Huangshan region [50]. This method fixes the data within the interval (0,1). For positive indicators, the larger the value, the closer the standardized value is to 1; for negative indicators, the larger the value, the closer the standardized value is to 0. The standardized matrix of evaluation indicators for factors influencing ecological space configuration optimization in the Greater Huangshan region is


$$\begin{array}{c} \left\{ \;\;\;\begin{array}{c} {\text{S}}_{ij}^{+}=\frac{s_{ij}-\text{min}\{s_{ij}\}}{\text{max}\{s_{ij}\}-\text{min}\{s_{ij}\}}\\ {\text{s}}_{ij}^{-}=\frac{\text{max}\{s_{ij}\}-s_{ij}}{\text{max}\{s_{ij}\}-\text{min}\{s_{ij}\}} \end{array}\right.\; \end{array}$$ (1)

In the formula: ${\text{s}}_{\text{ij}}^{+}$、${\text{s}}_{\text{ij}}^{-}$ are the standardized positive and negative indicators for the i-th indicator in the j-th year, respectively; i = 1,2,…,m,m (where mis the number of indicators); j = 1,2,…,n,n(where n is the number of years).

(2) Determining Indicator Weights and Constructing a Weighted Decision Matrix:

The entropy weight method is used to determine indicator weights, and a weighted normalized evaluation matrix (Y) is constructed by integrating entropy weights. The weight vector of each indicator is established T=(t1,t2,…,tn), and the weighted normalized matrix is


$$𝐘=S_{ij}\times 𝐓=[\begin{array}{cccc} {\text{y}}_{11} & {\text{y}}_{12} & \cdots & {\text{y}}_{1n}\\ {\text{y}}_{21} & {\text{y}}_{22} & \cdots & {\text{y}}_{2n}\\ \vdots & \vdots & \vdots & \vdots \\ {\text{y}}_{t1} & {\text{y}}_{t2} & \cdots & {\text{y}}_{tn} \end{array}]$$ (2)

Note: “ + ” indicates that the corresponding indicator is a positive indicator, where a larger numerical value represents a better evaluation result; “-” indicates that the corresponding indicator is a negative indicator, where a smaller numerical value represents a better evaluation result.

(3) Determining Positive and Negative Ideal Solutions:

The max ${\text{Y}}_{\text{ij}}$ of the i-th indicator in the j-th year is set as the positive ideal solution;the min ${\text{Y}}_{\text{ij}}$ of the i-th indicator in the j-th year is set as the negative ideal solution, with ${\text{Y}}^{-}$ designated as the negative ideal solution. The expression is:


$$\begin{array}{c} \begin{array}{cc} Y^{+}\; & \;\;\;\;=\left(\text{max}(1\leq j\leq n)y_{ij}|i=1\text{,}2\text{,}\cdots \text{,}n\right)\\ & \;\;\;\;\;\;\;\;\;\;=\left(y_{1}^{+}\text{,}y_{2}^{+}\text{,}\cdots \text{,}y_{n}^{+}\right)\;\; \end{array} \end{array}$$ (3)

$$\begin{array}{c} \begin{array}{cc} Y^{-}\; & \;\;\;\;=\left(\text{min}(1\leq j\leq n)y_{ij}|i=1\text{,}2\text{,}\cdots \text{,}n\right)\\ & \;\;\;\;\;\;\;\;\;\;\;\;\;\;\;=\left(y_{1}^{-}\text{,}y_{2}^{-}\text{,}\cdots \text{,}y_{n}^{-}\right)\;\; \end{array} \end{array}$$ (4)

In the formula: ${\text{Y}}_{\text{ij}}$ is the value of the i-th indicator in the j-th year in the weighted decision evaluation matrix V; ${\text{Y}}_{\text{i}}^{+}$、${\text{Y}}_{\text{i}}^{-}$ are the positive and negative ideal solutions for the i-th indicator, respectively.

(4) Calculating Distances:

The distances from indicators for factors influencing ecological space configuration optimization in the Greater Huangshan region to the positive and negative ideal solutions are


$$R_{i}^{+}=\sqrt{\sum_{i=1}^{n}(y_{ij}-y^{+}}{)}^{2}$$ (5)

$$R_{i}^{-}=\sqrt{\sum_{i=1}^{n}(y_{ij}-y^{-}}{)}^{2}$$ (6)

(5) Calculate the closeness (Ci):

Its expression is


$$c_{i}=\frac{{\text{R}}_{\text{i}}^{-}}{{\text{R}}_{i}^{+}+{\text{R}}_{\text{i}}^{-}}\text{\textbackslash{} }$$ (7)

Whereas: ${\text{C}}_{\text{i}}$∈[0,1], the closer it is to the ideal level of the influencing factors for ecospatial grouping optimization in the Greater Huangshan region during the first year, the larger its value.

According to the size of the relative posting progress in different years, the level of ecological spatial grouping optimization influencing.

## 3. Results and analysis

### 3.1. Comprehensive evaluation of ecological space in the Greater Huangshan Region

**Fig 3:**
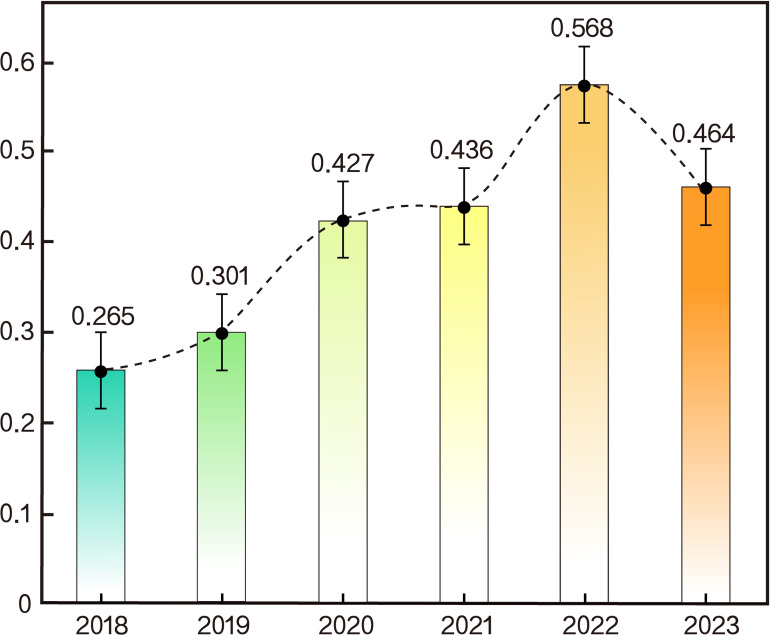
Line Chart of the Greater Huangshan Region, 2018–2023.

From 2018 to 2023, the comprehensive index of factors influencing ecological space configuration optimization in the Greater Huangshan region increased from 0.265 to 0.464 (Table 2). Using the DPSIR-TOPSIS model to analyze the 2018–2023 current status data of the Greater Huangshan region, a comprehensive evaluation index diagram oRanking Resultf factors influencing ecological space configuration optimization was obtained (Figure 3). As shown in the charts, the comprehensive index of factors influencing ecological space configuration optimization in the Greater Huangshan region shows an upward trend with minor fluctuations, which can be specifically divided into three stages:

**Table 2:** Relative Closeness in the Greater Huangshan Region from 2018 to 2023.

Year	Distance to Negative Ideal Solution	Distance to Negative Ideal Solution	Relative ClosenessC	Ranking Result
2018	0.18	0.065	0.265	6
2019	0.164	0.071	0.301	5
2020	0.144	0.107	0.427	4
2021	0.14	0.108	0.436	3
2022	0.111	0.145	0.568	1
2023	0.145	0.126	0.464	2

[1] Minor Upward Trend Phase: From 2018 to 2019, the index of factors influencing ecological space configuration optimization in the Greater Huangshan region increased from 0.265 in 2018 to 0.301 in 2019. Although the growth was modest, the overall ecological space showed a rising trend. The main reason is that since the “13th Five-Year Plan,” Anhui Province has actively implemented national policies and promulgated Implementation Opinions of the Anhui Provincial Party Committee and People’s Government on Comprehensively Strengthening Ecological Environment Protection and Firmly Winning the Pollution Prevention and Control War. In 2019, the Greater Huangshan region implemented “Five Control Measures” and deepened “Five Governance Measures” to effectively improve environmental quality.
[2] Significant Improvement Phase: In terms of indicators, the index of factors influencing ecological space configuration optimization in the Greater Huangshan region showed a substantial upward trend from 2019 to 2021, increasing from 0.301 in 2019 to 0.436 in 2021.The main reason is that 2020 was the concluding year of the “13th Five-Year Plan” and the decisive year for building a moderately prosperous society in all respects. Anhui Province deeply studied and implemented Xi Jinping Thought on Ecological Civilization, adhering to high-quality development. The Greater Huangshan region thoroughly advanced the pollution prevention and control campaign, achieving continuous and significant improvements in ecological and environmental quality. A secondary reason is that the sudden outbreak of the COVID-19 pandemic in 2020 severely hit the tourism industry. With the reduction in the scale of the tourism consumption market, the ecological environment space index of the Greater Huangshan region continued to improve.
[3] Fluctuating Upward Phase: From 2021 to 2023, the index of factors influencing ecological space configuration optimization in the Greater Huangshan region exhibited a fluctuating upward trend. First, there was a significant increase, rising from 0.436 in 2021 to 0.568 in 2022. The reason was that due to the COVID-19 pandemic, tourist numbers remained at a low level for an extended period, allowing the natural ecological environment in the Greater Huangshan region to recover significantly during this period. Reduced human activities alleviated environmental pressure, while enhanced ecological self-recovery capacity drove the rapid improvement of the ecological space index from its initial lower level. Subsequently, from 2022 to 2023, the index declined from 0.568 to 0.464. Following adjustments to COVID-19 prevention and control policies, regional economic activities quickly resumed. Factors such as accelerated industrial growth, increased population density, and rising land development demands led to a substantial increase in resource consumption, imposing new pressures on the ecological environment and causing the ecological space index to decline accordingly.

Therefore, the ecological space index in the Greater Huangshan region generally shows an upward trend with minor fluctuations, indicating a steady and positive overall development.

### 3.2. Characteristics of spatio-temporal difference distribution in the Greater Huangshan region

From 2018 to 2023, the ecological space configuration optimization in the Greater Huangshan region showed a fluctuating upward trend, with the overall level continuing to develop in a healthy and sustainable direction.

**Fig 4:**
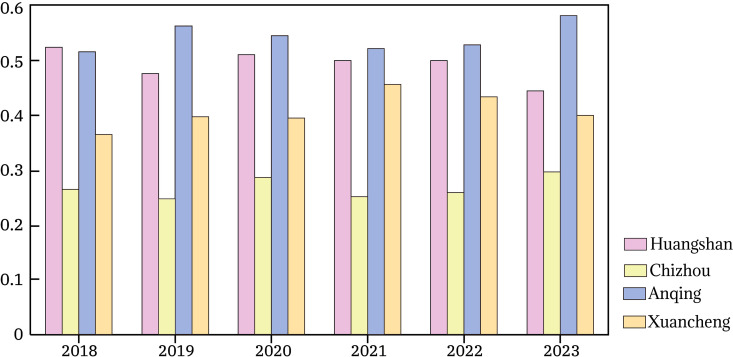
Ecological Indices Closeness Ranking,2018–2023.

At the temporal level, the ecological space configuration optimization levels of the four cities showed a trend of fluctuating growth year by year, as shown in Table 3. It exhibited a relatively obvious three-stage characteristic, as shown in Fig 4: from 2018 to 2019, the ecological space levels of cities in the Greater Huangshan region showed a modest upward trend, and the differences between cities gradually narrowed. From 2020 to 2021, except for Xuancheng City, all showed a slight decline. The main reason was that after the COVID-19 pandemic, cities entered a stage of rapid development. The growth in urbanization levels, urban population density, and tourist numbers placed significant pressure on the ecological space in the Greater Huangshan region.

**Table 3:** Closeness Ranking of ecological space Indices in Cities of the Greater Huangshan Region, 2018–2023.

Closeness Ranking of ecological space Indices in Cities of the Greater Huangshan Region								
Cities	2018	2019	2020	2021	2022	2023	Mean Value	Mean Ranking
Huangshan	0.519	0.476	0.516	0.501	0.498	0.44	0.492	2
Chizhou	0.268	0.245	0.28	0.249	0.255	0.292	0.265	4
Anqing	0.515	0.565	0.544	0.515	0.524	0.571	0.539	1
xuancheng	0.359	0.394	0.394	0.454	0.427	0.397	0.404	3

Overall, except for Huangshan City, all showed a modest upward trend. This is mainly due to the government's increased financial support and governance efforts for ecological space under the high-quality development strategic goals, leading to a continuously narrowing gap in ecological space levels among cities.

**Fig 5:**
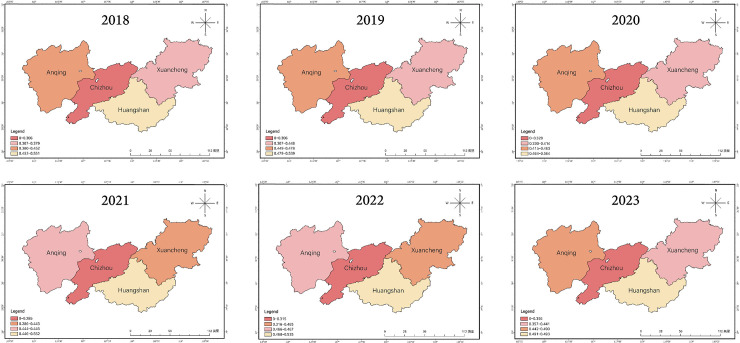
Spatial Distribution Map of Cities,2018–2023.

At the spatial level, significant differences exist in the ecological space indices among cities in the Greater Huangshan region, as shown in Figure 5. From a long-term perspective, Anqing City has performed the most prominently overall, achieving a relatively balanced approach to ecological protection and development. Huangshan City follows closely behind: relying on its rich cultural and ecological resources, its ecological space index has consistently remained at a high level. Xuancheng City has shown stable overall performance, particularly demonstrating positive improvement in recent years as the potential of its ecological tourism and agricultural development has been gradually unleashed. Although Chizhou City’s index is relatively low, it has shown a sustained improvement trend in recent years through the gradual strengthening of ecological protection measures. Overall, the gap in ecological space quality among cities in the region is gradually narrowing, reflecting a positive trend of collaborative development.

### 3.3. Ecological space subsystem Index in the Greater Huangshan Region

Each subsystem displays certain differences in its change trends, as illustrated in Table 4. Specifically:

**Table 4:** Evaluation Results of Each Subsystem in the Greater Huangshan Region, 2018–2023.

Year	Drive D	Pressure P	State S	Impact I	Response R
2018	0.1852	0.2047	0.1755	0.2024	0.2323
2019	0.1846	0.1874	0.1853	0.2038	0.2388
2020	0.1808	0.2006	0.1799	0.2225	0.4387
2021	0.2072	0.1869	0.1824	0.2063	0.2172
2022	0.1740	0.2175	0.1840	0.2161	0.2085
2023	0.2037	0.2217	0.1650	0.1858	0.2238

(1) Driving Force Subsystem

**Fig 6:**
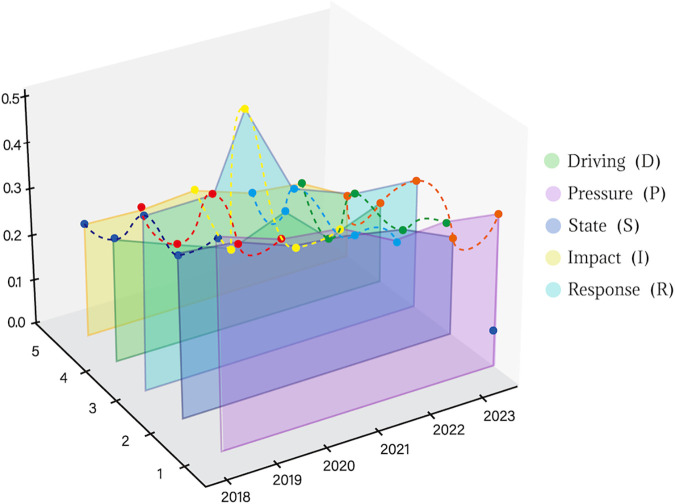
Line Chart of Subsystem Evaluation Results,2018–2023.

From 2018 to 2023, the driving force index in this region showed a fluctuating upward trend, as shown in Figure 6, mainly influenced by population density, GDP, and urbanization rate. In the early stage, due to economic growth and urbanization, the index increased; later, affected by the pandemic, population growth slowed and economic activities weakened, causing a brief decline in the index. In the later stage, with economic recovery and rising household incomes, the index resumed its upward trend. Overall, the regional social development foundation has gradually strengthened, but the growth pace remains relatively gentle.

(2) Pressure Subsystem

Pressure is a key factor affecting configuration optimization in the Greater Huangshan region. From 2018 to 2023, the pressure indicator layer index in the Greater Huangshan region first rose and then fell. In the early stage, the pressure index increased due to rising energy consumption and total tourist numbers, which intensified pressures on the region. Rapid economic growth often came at the expense of the environment.With the profound implementation of ecological civilization construction, the concept that “lucid waters and lush mountains are invaluable assets” has taken root in people's minds. Especially after 2021, government investment in environmental protection increased significantly, promoting continuous positive changes in the environment. Responding to national policies, the Greater Huangshan region began to improve its ecological environment, leading to improvements in the pressure sub-indicator layer index. However, overall, the ecological space in this region still faces significant pressure, indicating that the Greater Huangshan region needs to continue increasing policy and financial support to promote sustained positive environmental changes.

(3) State Subsystem

The state is shaped by the influence of pressure and driving forces. From 2018 to 2023, the state indicator layer index in the Greater Huangshan region showed a declining trend, which indicates a marked improvement in the ecological space status of the region. 2019 was a critical turning point: due to the impact of the COVID-19 pandemic and the continuous advancement of national ecological environmental protection policies, the state indicator layer index in the Greater Huangshan region began to decline steadily. This suggests that factors such as increased green space area have driven the ecological space toward improvement.

However, overall, the state subsystem in the Greater Huangshan region still remains at a moderate level, indicating that the situation regarding ecological space configuration optimization remains relatively severe.

(4) Impact Subsystem

The impact index shows a fluctuating upward trend, indicating a two-way interaction between ecological protection and socioeconomic development.This is mainly because since the “13th Five-Year Plan” period, the Greater Huangshan region has responded to policy initiatives by increasing investment in environmental governance, leading to continuous improvements in the ecological environment. Therefore, the impact layer index has shown an upward trend. From 2022 to 2023, it dropped from 0.1840 to 0.1650, primarily due to rapid economic development after the COVID-19 pandemic and the continuous rise in urbanization rates in the Greater Huangshan region.

With continuous population inflow, per capita land area and per capita urban construction land area have gradually decreased, causing a short-term decline in the impact index. In the long term, ecological environmental protection has exerted a positive promotional effect on the social and economic development of the Greater Huangshan region, and the degree of influence has become increasingly significant.

(5) Response Subsystem

From 2018 to 2023, the ecological space response indicator layer index in the Greater Huangshan region continued to rise, indicating that the effectiveness of environmental protection policies has gradually become evident. The main reason is that the local government has continuously invested in energy conservation, environmental protection, and increased spending on environmental governance, steadily improving the ecological space. The response subsystem reflects government regulation and policy prioritization: the index growth shows that various policies formulated by the Greater Huangshan regional government have achieved remarkable results in protecting ecological space. Meanwhile, the increase in the added value of the tertiary industry has provided a solid foundation, further accelerating the optimization of the response subsystem.

However, the overall improvement remains modest, suggesting that the region still needs to explore and actively implement effective policies to further enhance ecological space.

## 4. Discussion

### 4.1. Methodological innovations and limitations

Currently, there is no unified ES evaluation criteria [51,52]. This study constructs an ecological space configuration optimization (ES-GO) evaluation system based on the DPSIR model (Driving Force-Pressure-State-Impact-Response), covering five dimensions: economy, society, technology, resources, and environment. An entropy weight-TOPSIS composite model is used to achieve objective weighting and priority ranking, which helps measure ES-GO more scientifically and effectively.This comprehensive approach allows for a deeper understanding of the diversity and complexity of influencing factors on ecological space in urban agglomerations, revealing the causal chain of ecological space evolution (e.g., rising urbanization rate → increased ecological pressure → government regulatory response) [53–55]; Unlike previous studies focusing on specific cities [56], this research uses data from four cities in the Greater Huangshan Region (DHR) during 2018–2023 to reveal the nonlinear characteristics of ecological space changes in DHR, providing a basis for dynamic regulation. The evaluation results can objectively and scientifically reflect the current ecological and environmental conditions, offering data support and references for formulating ecological and environmental protection policies and strategies [57]. Additionally, the research methods and evaluation index system constructed in this study can be adapted to sustainability assessments of other urban agglomerations, demonstrating universal value.

In addition, although this study references many existing research findings when selecting influencing factor indices for ecological space configuration optimization in the Greater Huangshan region, limitations in data collection channels, obstacles to open data sharing, and differences in data quality standards have led to the failure to evaluate certain ecological indicators.Examples include the region’s ecosystem health, water quality, and animal habitat area [58,59]. The evaluation index system still requires improvement in follow-up research.

### 4.2. Policy recommendations

Given that Anhui Province is fully promoting the construction of the Greater Huangshan Region, the demand for environmental carrying capacity and resource resilience in this area is expected to continue increasing. To promote sustainable development, maintain long-term stability of ecological space, and achieve coordinated economic, social, and ecological development, the following recommendations are proposed:

First, increase financial support for the development of ecological space in the Greater Huangshan Region. Governments should reasonably allocate expenditures to ensure the effective use of funds and steadily advance the implementation of ecological space configuration optimization (ES-GO) projects in the region [60].

Second, implement differentiated regional regulation based on the specific conditions of each city in the Greater Huangshan Region and the changing trends of influencing factors for ES-GO.Chizhou City has a relatively single economic structure, where government supervision and guidance are crucial—financial and policy support should be provided [61]. For example, efforts should be made to expand opening-up, attract investment, and vigorously develop emerging industries and characteristic industries.In contrast, Anqing and Xuancheng Cities have enormous potential in developing eco-tourism and characteristic industries; they should fully utilize their resources, leverage their unique advantages, and pursue high-quality development.As for Huangshan City, which is naturally endowed with strong brand recognition the government should strive to upgrade traditional sightseeing tourism to modern leisure, vacation, and wellness tourism. This can be achieved by continuously enhancing the city’s unique natural environment, enriching biodiversity, and upgrading both hardware and software quality to extend tourists’ stay.

Finally, increase investment in technology to improve resource utilization efficiency.Measures such as upgrading harmless treatment capabilities, promoting clean production, and vigorously implementing energy conservation and emission reduction should be prioritized to alleviate pressure on ecological space.

## 5. Conclusions

Through a comprehensive analysis of the influencing factor indices and subsystems of ecological space configuration optimization (ES-GO) in the Greater Huangshan Region from 2018 to 2023, the following conclusions are drawn:

(1) Comprehensive Analysis of Ecological Space Index in the Greater

The weight calculation results of the indicators show that production-living factors and population factors have a more significant impact on ES-GO. For example, the urbanization rate and the number of private vehicles owned are core sources of ecological pressure. Resource factors and social factors, however, exert more fundamental and extensive influences on ES-GO. For instance, forest coverage rate and air quality compliance rate contribute most to ecological restoration, highlighting the foundational role of “natural resources.”

(2) Analysis Based on DPSIR Subsystems

The driving force (D) showed the fastest growth rate, but improvements in pressure (P) and state (S) lagged, indicating that the path dependence of “prioritizing development over protection” has not been fundamentally reversed. In the context of ecological space protection, there still exists a high-input, high-consumption extensive development pattern. The response subsystem exhibited a sustained downward trend after reaching a phased peak, suggesting a transmission lag in the “policy response-pressure mitigation” mechanism. Overall, both the pressure subsystem and state subsystem still have substantial room for improvement.

(3) Characteristics of Spatial-Temporal Difference Distribution

From the temporal characteristics dimension: The ecological space index of the Greater Huangshan Region showed a slight fluctuating upward trend, but experienced a short-term decline in 2022 due to post-pandemic economic recovery demands. Overall, ecological space configuration optimization in the Greater Huangshan Region demonstrated good stability during 2018–2023.From the spatial characteristics dimension: The largest differences were observed between Anqing City and Chizhou City, reflecting more severe “protection-development” contradictions in cities with single-industry structures. Huangshan City and Xuancheng City ranked second and third, respectively, with a small gap between them. Although Huangshan City is endowed with abundant ecological resources, its index declined to 0.44 in 2023, reflecting the ecological impact of tourism overloading. Xuancheng City, however, achieved steady improvements through eco-agriculture and wetland restoration.The data indicate that despite ecological protection measures taken by all cities, differences in development models have led to divergent restoration outcomes.

This study reveals the core contradiction of “policy response lagging behind development pressure” by analyzing the ecological space configuration optimization mechanism in the Greater Huangshan Region from multiple dimensions. In the future, efforts should be anchored in the “pressure-response” dynamic balance to build a more resilient spatial governance system, providing scientific guidance for realizing the transformation path of “lucid waters and lush mountains are invaluable assets.”
